# Adjuvant VACcination against HPV in surgical treatment of Cervical Intra-epithelial Neoplasia (VACCIN study) a study protocol for a randomised controlled trial

**DOI:** 10.1186/s12885-020-07025-7

**Published:** 2020-06-09

**Authors:** R. L. O. van de Laar, W. Hofhuis, R. G. Duijnhoven, S. Polinder, W. J. G. Melchers, F. J. van Kemenade, R. L. M. Bekkers, H. J. Van Beekhuizen

**Affiliations:** 1 Department of Gynecologic Oncology, Erasmus MC Cancer Institute, University Medical Centre Rotterdam, PO Box: 2040, 3000 CA Rotterdam, The Netherlands; 2 Department of Obstetrics and Gynaecology, Franciscus Gasthuis, PO Box: 10900, 3004 BA Rotterdam, The Netherlands; 3 Clinical trials unit of the Dutch Society for Obstetrics and Gynecology, Amsterdam UMC, University of Amsterdam, Meibergdreef 9, 1105 AZ Amsterdam, Netherlands; 4 Department of Public Health, Center for Medical Decision Sciences, Erasmus MC- University Medical Centre Rotterdam, Rotterdam, The Netherlands; 5 Department of Medical Microbiology, Radboud University Medical Centre, PO Box 9101, 6500 HB Nijmegen, the Netherlands; 6 Department of Pathology, Erasmus MC, University Medical Centre Rotterdam, Rotterdam, 3000 CA The Netherlands; 7 Department of Obstetrics and Gynecology, Catharina Hospital, PO Box 1350, 5602 ZA Eindhoven, the Netherlands; 8 GROW School for Oncology and Developmental Biology, Maastricht University, Eindhoven, the Netherlands

**Keywords:** Cervical intraepithelial neoplasia (CIN), HSIL, Human papillomavirus (HPV), Loop electrosurgical excision procedure (LEEP), HPV-vaccination, Recurrence, Persistence

## Abstract

**Background:**

Cervical cancer is caused by Human Papilloma viruses (HPV) and is preceded by precursor stages: Cervical Intraepithelial Neoplasia (CIN). CIN is mostly found in women in their reproductive age and treated with a Loop Electrosurgical Excision Procedure (LEEP). The recurrence or residual disease rate after treatment is up to 17%. These women have a lifelong increased risk of recurrent CIN, cervical cancer and other HPV related malignancies. Furthermore, LEEP treatments are associated with complications such as premature birth. Limited data show that prophylactic HPV vaccination at the time of LEEP reduces recurrence rates, therefore leading to a reduction in repeated surgical interventions and side effect like preterm birth.

The primary study objective is to evaluate the efficacy of the nonavalent HPV vaccination in women with a CIN II-III (high-grade squamous intraepithelial lesion (HSIL) lesion who will undergo a LEEP in preventing recurrent CIN II-III after 24 months.

**Methods:**

This study is a randomised, double blinded, placebo controlled trial in 750 patients without prior HPV vaccination or prior treatment for CIN and with histologically proven CIN II-III (independent of their hrHPV status) for whom a LEEP is planned. Included patients will be randomised to receive either three injections with nonavalent (9 HPV types) HPV vaccine or placebo injections (NaCL 0.9%) as a comparator. Treatment and follow-up will be according the current Dutch guidelines. Primary outcome is recurrence of a CIN II or CIN III lesion at 24 months. A normal PAP smear with negative hrHPV test serves as surrogate for absence of CIN. At the start and throughout the study HPV typing, quality of life and cost effectiveness will be tested.

**Discussion:**

Although prophylactic HPV vaccines are highly effective, little is known about the effectivity of HPV vaccines on women with CIN. Multiple LEEP treatments are associated with complications. We would like to evaluate the efficacy of HPV vaccination in addition to LEEP treatment to prevent residual or recurrent cervical dysplasia and decrease risks of repeated surgical treatment.

**Trial registration:**

Medical Ethical Committee approval number: NL66775.078.18. Affiliation: Erasmus Medical Centre. Dutch trial register: NL 7938. Date of registration 2019-08-05.

## Background

Worldwide, cervical cancer is diagnosed annually in more than 500,000 women and is still a major health problem among women worldwide [1]. Cervical cancer is preceded by Cervical Intraepithelial Neoplasia (CIN) of the cervix and caused by high risk Human Papilloma Viruses (hrHPV) [2]. CIN is subdivided in three groups: CIN I mild dysplasia, also known as low grade squamous intraepithelial lesion (LSIL). CIN II is mild dysplasia and CIN III severe dysplasia, both also known as high-grade squamous intraepithelial lesion (HSIL). Persistent hrHPV infection is a prerequisite to develop cervical cancer. About 80% of women will be infected with HPV during their life and most women are able to clear the HPV infections. However, approximately 20% of these women have detectable transient infections and a fraction will subsequently develop to cervical cancer if not treated for these precursor lesions [3]. With adequate screening and treatment of CIN, cervical cancer can be prevented [4].

The most commonly used method to treat CIN II-III is Loop Electrosurgical Excision Procedure (LEEP) to prevent possible progression to invasive cancer. Data on recurrent disease after LEEP vary in the literature. Up to 17% of women treated for CIN II-III have residual or may develop recurrent CIN II-III [5, 6]. Treatment is associated with side effects such as hemorrhage, infection, and stenosis of the cervix, and as well as adverse pregnancy outcomes, such as premature rupture of membranes and premature birth. Re-excision is needed in case of recurrence with an increased risk for adverse events. Especially the adverse obstetrical outcomes are higher after multiple treatments [7–9]. Women treated for CIN also have an increased risk of developing cervical, vaginal and vulvar cancer compared to patients with normal primary smear test results [10–12]. To prevent HPV-related diseases, eradication is preferable to treating recurrences repeatedly [13]. Recurrence can be monitored by HPV testing.

Many studies have proven the efficacy and safety of the prophylactic HPV vaccine against the development of cervical intraepithelial neoplasia in HPV naïve women [14–16]. In 2013, Kang et al. reported retrospective data that demonstrated possible prevention of CIN II-III recurrence after LEEP treatment when treatment was combined with quadrivalent HPV vaccination. This study showed that 5 patients (2.5%) in the vaccinated group (197 patients) and 18 patients (8.5%) in the unvaccinated group (211 patients) developed recurrent disease after LEEP (relative risk 0.17, 95%CI 0.08–0.36; *P* < 0.001) related to the vaccine HPV types. Irrespective of HPV vaccine type the recurrence rate was 2.5% (9/360) in the vaccinated group versus 7.2% (27/377) in the unvaccinated group. Multivariate analysis showed that no vaccination after LEEP was an independent risk factor for recurrent CIN II-III (HR = 2.8; 95% confidence interval 1.3–6.0; *P* < 0.01) [17]. Previously, Joura et al. concluded from post hoc analysis, women receiving prophylactic HPV vaccine in a randomized setting and treated for HPV related diseases (cervical, vaginal, vulvar) are less likely to develop recurrent lesions compared to those not vaccinated [18]. There is increasing evidence of an additional vaccine effect after treatment of clinical HPV related anogenital, dermal and oropharyngeal diseases [17, 19–22]. Hypothetically, an increased immune response with increasing levels of antibodies after HPV vaccination might explain the effect. In addition, there ought to be protection against de novo HPV infections [23, 24]. A randomised controlled trial is lacking on this subject.

In this study, we will compare HPV vaccination with the nonavalent HPV vaccine (types 6, 11, 16, 18, 31, 33, 45, 52, and 58) versus placebo (physiological salt solution) vaccination subsequently to LEEP treatment in women without previous treatment for CIN or HPV vaccination. This study is a randomised, double blinded, placebo controlled trial to evaluate the efficacy of the nonavalent HPV vaccine in preventing recurrent CIN II-III after 24 months.

## Methods

### Setting and study design

The VACCIN study is a randomised multicenter, double blinded, placebo controlled trial in female patients with histological diagnosed CIN II or CIN III and treated with LEEP. Recruitment of participants will be done from colposcopy clinics in participating centers. After histologic diagnosis of CIN II-III for which a LEEP is necessary, patients will be counselled for participating in this study.

In the Netherlands most women with CIN are detected via population based cervical cancer screening starting at the age of 30 years.

In addition, general practitioners may refer women with intermenstrual or postcoital bleeding. This also includes women younger than those in the national screening program for cervical cancer. When abnormal Pap smears are found, they are referred for colposcopy.

### In- and exclusion criteria

Women are eligible for participation if they are eighteen years old or above, histologically proven CIN II or III and will be treated with a LEEP. Inclusion must be within four weeks after LEEP. Exclusion criteria are: a prior HPV vaccination, invasive carcinoma, immune-compromised women, pregnant women, prior treatment for CIN-lesions, insufficient understanding of the Dutch language or allergic to vaccine components.

### Randomisation, blinding and treatment allocation

Randomisation will be performed through the secure web-based interface Castor EDC (Castor Electronic Data Capture, available at: https://castoredc.com). Patients will be randomised to either active study medication, or placebo in a 1:1 ratio. Randomisation will be stratified by age, categorized as being 18 to 29 years, 30 to 44 years, or 45 years and older. Women aged 18 to 29 years are not eligible for routine screening under the national screening program. The second stratum includes women of 30 to 44 years who are of reproductive age and invited for routine testing under the national screening program. The third stratum women of 45 years and older, who are less likely to be fertile. Randomisation will use random permuted blocks of sizes 2 and 4.

Both doctor and patient will be blinded for treatment allocation. The allocated medication will be prepared and distributed by the hospital pharmacy as syringes with identical appearance.

### Study objectives and outcome

The primary objective is:

The efficacy of nonavalent HPV vaccination in women with a CIN II-III lesion who underwent a LEEP in preventing recurrent CIN II-III after at 24 months follow-up.

The secondary objectives are:

1. The recurrence of CIN I-II-III at 6, 12 and 24 months.
2. The effect of HPV vaccination on HPV DNA presence at 6 and 24 months after LEEP.
3. The effect of HPV vaccination on Pap-smear results.
4. Number of LEEP for recurrent CIN.
5. Cost-effectiveness analysis from a societal perspective: intramural and extramural medical costs questionnaire (iMCQ) and productivity cost questionnaire (iPCQ) will be performed at the start of the study, at 2 months and after 24 months.
6. Quality of life will be assessed with quality of life questionnaire: Euroqol 5D-5 L at the start of the study, at 2 months, at 6 months and after 24 months.
7. Side effects and adverse events. One week after each vaccination, a telephonic interview will be performed to ask for side effects after the injection.

The tertiary and long-term objectives are:

1. Cytology result after 5 years by retrieving HPV-test and Pap smear results from the national database of the population based cervical cancer screening program.
2. Cytology result after 10 years by retrieving HPV-test and Pap smear results from the national database of the population based cervical cancer screening program.
3. Vaccination effect on premature delivery and obstetrical complications by comparing the results to a national database on childbirth data.

### Interventions

Patients eligible for participation will be counselled and receive written information. All patients must provide written informed consent prior to participation. After written consent patients will be randomised to either

- Nonavalent HPV-vaccine (Gardasil-9®) or
- Placebo vaccine (physiological salt solution for injections)

**Fig. 1:**
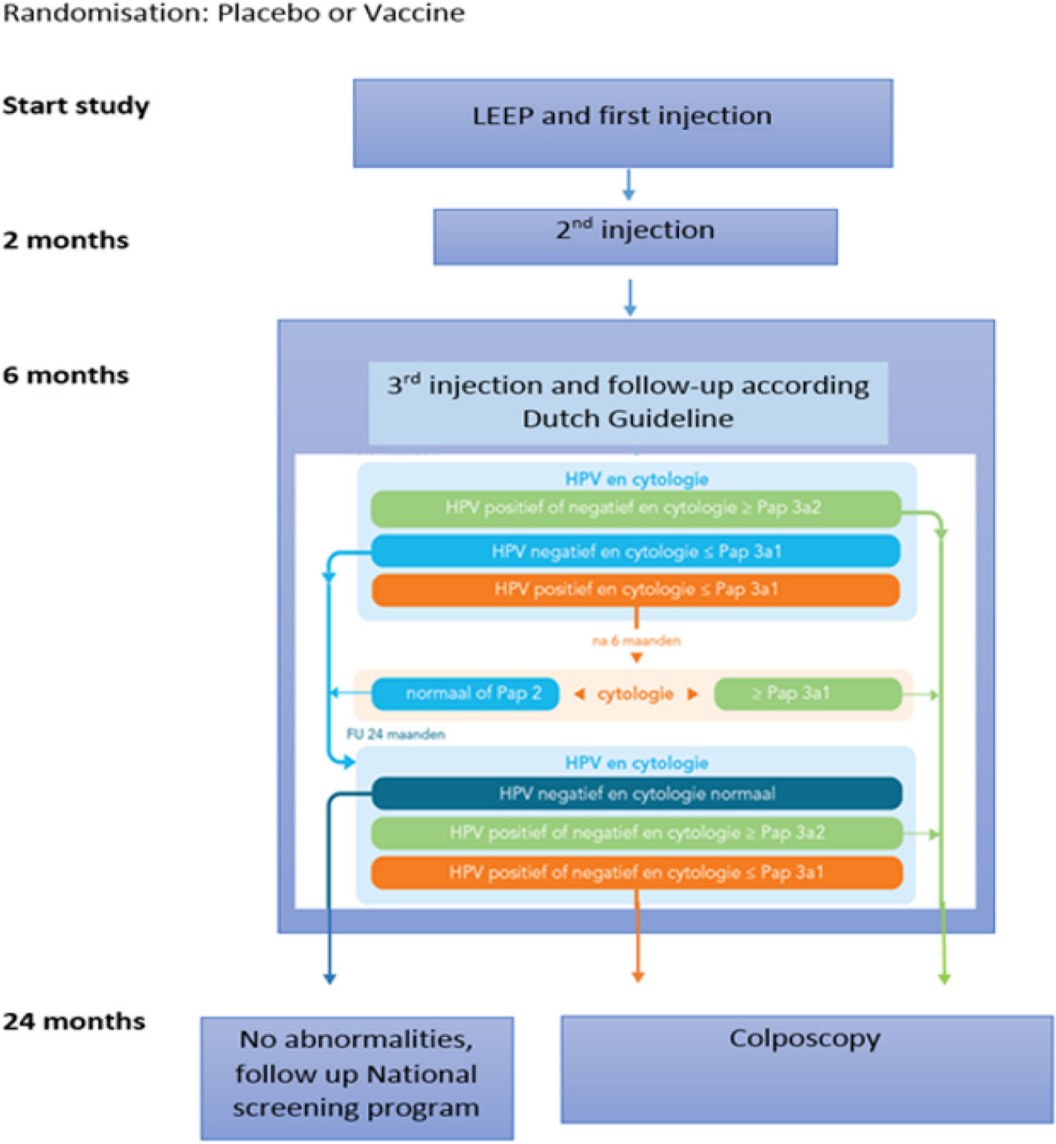
Flowchart study design follow-up

The LEEP should be performed according to guideline for regular care. Preferably, at the day of LEEP the first vaccination should be administered. The first vaccination should be administered at least within 4 weeks after the LEEP. This time window was chosen in particular for logistical reasons, for example with the see and treat method. The second dose should be administered at least one month after the first dose and the third dose should be administered at least 3 months after the second dose. All three doses should be given within a 1-year period. Although studies do not indicate adverse pregnancy outcomes, patients will be asked to use reliable contraceptive during the vaccination period (6 months). Follow-up will be according the Dutch guideline for cervical and vaginal dysplasia (CIN, AIS and VAIN 2015) [25], (see Fig. 1: Flowchart study design). One week after each vaccination, telephonic interview is performed to ask for side effect of the injection.

### Sample size calculations

The sample size calculations have been based on the only publication available at the time of preparing this trial. This retrospective cohort study describes an incidence of 2.5% in women treated with quadrivalent HPV vaccine, against 8.5% in the untreated group (Kang et al., 2013). With a target power of 0.8 and an alpha of 0.050, a total number of 646 patients needs to be recruited into the study if the incidence of CIN II-III at two-year follow-up is 3% in the group receiving active treatment, and 8% in the group receiving placebo. Clinical practice shows that the proportion of women who do not attend follow-up visits in this population is high. Therefore, a loss to follow-up is anticipated of 10 to 15%. To compensate for such attrition, the total number of patients that is to be recruited for the study is rounded to 750 patients in total.

### Data collection

All data will be collected using an eCRF and by means of electronic questionnaires. The eCRF and questionnaire data will be collected and stored using Castor EDC, which is also used for randomisation. Subject numbers will be assigned sequentially to subjects enrolled in the study. Stored data is only accessible to the principal and coordinating investigator.

**Fig. 2:**
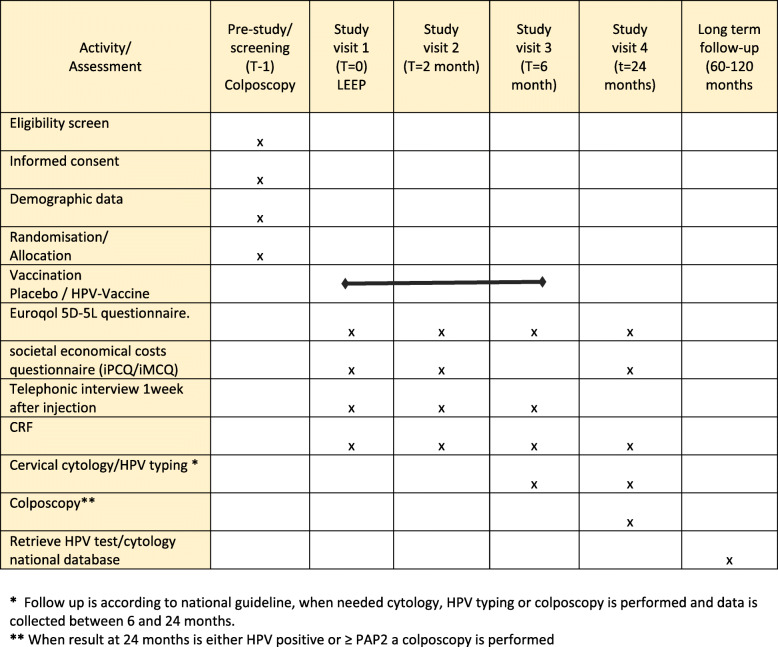
Schedule of enrolment, interventions, and assessments VACCIN-study. * Follow up is according to national guideline, when needed cytology, HPV typing or colposcopy is performed and data is collected between 6 and 24 months. ** When result at 24 months is either HPV positive or ≥ PAP2 a colposcopy is performed

The following data and outcome parameters are collected (see also Fig. 2):

- Patient characteristics; age, obstetrical history, contraceptive use, condom use complementary to contraceptive, HPV related history, smoking, family planning.
- Indication for testing, cytology results, KOPAC-classification, HPV genotype, biopsy result.
- Quality of life questionnaire (Euroqol 5D-5 L) and societal economical costs questionnaire (iPCQ, adjusted iMCQ).
- Initial Pap smear and HPV test results

1 week follow-up:

- Side effects of the injection, Result of LEEP (no CIN, CIN I, II or III) and LEEP margins (clear margins or CIN in margins).

2 months follow up:

- Side effects of the injection.
- QoL (Euroqol 5D-5 L) and societal economical costs questionnaire (iPCQ, adjusted iMCQ).

6 months follow-up:

- Cytology results and HPV genotype, QoL (Euroqol 5D-5 L) questionnaire.
- Side effects of the injection.

24 month follow-up:

- Cytology results and HPV genotype. When either test is abnormal, colposcopy will be performed and biopsies taken for histologic study endpoint.
- QoL and societal economical costs questionnaire (iPCQ, adjusted iMCQ).
- All other cytology test, colposcopy results, LEEP result and HPV genotype test between appointment 6 months and 24 months follow-up if appropriate.
- Pregnancy during follow-up with obstetrical outcome.

60 and 120 months follow-up:

- Collection of HPV test and Pap smear result via population screening program and sub sequential treatment.
- Collection of national database on childbirth data on subsequent pregnancies and the following pregnancy outcomes: gestational age, miscarriages, preterm rupture of membranes.

### Statistical analysis

The main analysis for the primary outcome testing the hypothesis of superiority of nonavalent vaccine as compared to placebo, for preventing CIN II-III recurrence after 24 months will be conducted using a χ^2^ test. Outcome measure for the primary outcome is a relative risk with 95% confidence interval.

Relative risks will also be estimated for secondary categorical outcomes, with 95% confidence intervals and χ^2^ or Fisher’s exact test for significance. Continuous data will be described using means with standard deviation, or medians with interquartile ranges; t-tests or Mann-Whitney U tests will be used as appropriate. Kaplan-Meier curves will be made for time between LEEP, vaccination with active or placebo and CIN II-III recurrence. The curves will be compared using the log-rank test. Quality of Life will be measured at every visit. They will be assessed with Euroqol 5D-5 L questionnaire. Patients from both groups will be analyzed using generalized linear mixed models.

The cost-effectiveness will be assessed by calculating the incremental cost-effectiveness ratio (ICER), defined as the difference in costs, divided by the average change in effectiveness of the vaccine versus a placebo following LEEP treatment. A Budget impact analysis (BIA) will be performed, in accordance with the principles for good practice for BIA, to address the financial stream of consequences in order to assess the affordability of offering the vaccine following LEEP treatment. BIA will compare the likely impact of replacing LEEP by HPV vaccination on national health plan budgets, on hospital level and on assurance level. From the viewpoint of the government, the broad societal perspective and the ‘budgetary framework for care’ will be highlighted. A valid framework (Markov model) will give insight into the budget consequences. We will also perform sensitivity analyses and a ‘most optimistic versus pessimistic’ scenario. If the vaccine has a preventive effect, it will be included in the new national guideline. As a result, the health care insurance can compensate this as regular care.

## Discussion

Prophylactic HPV vaccines are very effective in reducing premalignant lesions of the cervix in HPV negative young women and less so in women up to 25 years of age [26]. Less clear (i.e. only retrospective studies) are effects of adjuvant vaccination in context of CINII-III treatment in HPV positive women. The results of this prospectively randomized study will demonstrate whether adjuvant vaccination has a positive effect on recurrent CINII-III necessitating a second LEEP. This study will contribute to the understanding and treatment of HPV related diseases but is not sufficiently power for these endpoints. The strength of this trial is that it is a double blind- placebo controlled adequately power trial with long-term follow-up to measure effect on recurrent-relapsing CIN II-III.

## Data Availability

The datasets used and/or analyzed during the current study are available from the corresponding author on reasonable request.

## Abbreviations

CIN: Cervical Intraepithelial Neoplasia
LSIL: Low-grade squamous intraepithelial lesion
HSIL: High-grade squamous intraepithelial lesion
HPV: Human Papillomavirus
hrHPV: High risk Human Papillomavirus
LEEP: Loop Electrosurgical Excision Procedure
iPCQ: iMTA Productivity Cost Questionnaire
iMCQ: iMTA Medical Consumption Questionnaire

## References

[1] Arbyn M, Castellsague X, de Sanjose S, Bruni L, Saraiya M, Bray F (2011). Worldwide burden of cervical cancer in 2008. Ann Oncol.

[2] Schiffman M, Castle PE, Jeronimo J, Rodriguez AC, Wacholder S (2007). Human papillomavirus and cervical cancer. Lancet..

[3] Ho GY, Bierman R, Beardsley L, Chang CJ, Burk RD (1998). Natural history of cervicovaginal papillomavirus infection in young women. N Engl J Med.

[4] Nieminen P, Kallio M, Anttila A, Hakama M (1999). Organised vs. spontaneous pap-smear screening for cervical cancer: a case-control study. Int J Cancer.

[5] Kocken M, Helmerhorst TJ, Berkhof J, Louwers JA, Nobbenhuis MA, Bais AG (2011). Risk of recurrent high-grade cervical intraepithelial neoplasia after successful treatment: a long-term multi-cohort study. Lancet Oncol.

[6] van der Heijden E, Lopes AD, Bryant A, Bekkers R, Galaal K. Follow-up strategies after treatment (large loop excision of the transformation zone (LLETZ)) for cervical intraepithelial neoplasia (CIN): impact of human papillomavirus (HPV) test. Cochrane Database Syst Rev. 2015;1. 10.1002/14651858.CD010757.pub2 PMC6457759 25562623

[7] Kyrgiou M, Athanasiou A, Paraskevaidi M, Mitra A, Kalliala I, Martin-Hirsch P (2016). Adverse obstetric outcomes after local treatment for cervical preinvasive and early invasive disease according to cone depth: systematic review and meta-analysis. BMJ..

[8] Jin G, LanLan Z, Li C, Dan Z (2014). Pregnancy outcome following loop electrosurgical excision procedure (LEEP) a systematic review and meta-analysis. Arch Gynecol Obstet.

[9] Bjorge T, Skare GB, Bjorge L, Trope A, Lonnberg S (2016). Adverse pregnancy outcomes after treatment for cervical intraepithelial Neoplasia. Obstet Gynecol.

[10] Strander B, Andersson-Ellstrom A, Milsom I, Sparen P (2007). Long term risk of invasive cancer after treatment for cervical intraepithelial neoplasia grade 3: population based cohort study. BMJ..

[11] Ebisch RMF, Rutten DWE, IntHout J, Melchers WJG, Massuger L, Bulten J (2017). Long-lasting increased risk of human papillomavirus-related carcinomas and Premalignancies after cervical intraepithelial Neoplasia grade 3: a population-based cohort study. J Clin Oncol.

[12] Loopik DL, Ebisch RM, IntHout J, Melchers WJ, Massuger LF, Bekkers RL, et al. The relative risk of noncervical high-risk human papillomavirus-related (pre)malignancies after recurrent cervical intraepithelial neoplasia grade 3: a population-based study. Int J Cancer. 2019. 10.1002/ijc.32834 PMC7318313 31846057

[13] Lehtinen M, Baussano I, Paavonen J, Vanska S, Dillner J (2019). Eradication of human papillomavirus and elimination of HPV-related diseases - scientific basis for global public health policies. Expert Rev Vaccines..

[14] Arbyn M, Bryant A, Beutels P, Martin-Hirsch PP, Paraskevaidis E, Van Hoof E, et al. Prophylactic vaccination against human papillomaviruses to prevent cervical cancer and its precursors. Cochrane Database Syst Rev. 2011;2011(4). 10.1002/14651858.CD009069 PMC4176676 25267916

[15] Arbyn M, Xu L (2018). Efficacy and safety of prophylactic HPV vaccines. A Cochrane review of randomized trials. Expert Rev Vaccines.

[16] Group FIS (2007). Quadrivalent vaccine against human papillomavirus to prevent high-grade cervical lesions. N Engl J Med.

[17] Kang WD, Choi HS, Kim SM (2013). Is vaccination with quadrivalent HPV vaccine after loop electrosurgical excision procedure effective in preventing recurrence in patients with high-grade cervical intraepithelial neoplasia (CIN2-3)?. Gynecol Oncol.

[18] Oura Elmar A, Garland Suzanne M, Paavonen Jorma, Ferris Daron G, Perez Gonzalo, Ault Kevin A et al. Effect of the human papillomavirus (HPV) quadrivalent vaccine in a subgroup of women with cervical and vulvar disease: retrospective pooled analysis of trial data. BMJ. 2012;344:e1401. 10.1136/bmj.e1401 PMC3314184 22454089

[19] Pham CT, Juhasz M, Sung CT, Mesinkovska NA (2020). The human papillomavirus vaccine as a treatment for human papillomavirus-related dysplastic and neoplastic conditions: a literature review. J Am Acad Dermatol.

[20] Rosenberg T, Philipsen BB, Mehlum CS, Dyrvig AK, Wehberg S, Chirila M (2019). Therapeutic use of the human papillomavirus vaccine on recurrent respiratory Papillomatosis: a systematic review and meta-analysis. J Infect Dis.

[21] Dion GR, Teng S, Boyd LR, Northam A, Mason-Apps C, Vieira D (2017). Adjuvant human papillomavirus vaccination for secondary prevention: a systematic review. JAMA Otolaryngol Head Neck Surg.

[22] Ghelardi A, Parazzini F, Martella F, Pieralli A, Bay P, Tonetti A (2018). SPERANZA project: HPV vaccination after treatment for CIN2. Gynecol Oncol.

[23] Einstein MH, Schiller JT, Viscidi RP, Strickler HD, Coursaget P, Tan T (2009). Clinician's guide to human papillomavirus immunology: knowns and unknowns. Lancet Infect Dis.

[24] Velentzis LS, Brotherton JML, Canfell K (2019). Recurrent disease after treatment for cervical pre-cancer: determining whether prophylactic HPV vaccination could play a role in prevention of secondary lesions. Climacteric..

[25] Guideline CIS, AIS and VAIN 2015-11-12, Version: 1.0. https://www.oncoline.nl/cin-ais-en-vain..

[26] Drolet M, Benard E, Perez N, Brisson M, Group HPVVIS (2019). Population-level impact and herd effects following the introduction of human papillomavirus vaccination programmes: updated systematic review and meta-analysis. Lancet..

